# Characterization of an Autophagy-Related Gene *MdATG8i* from Apple

**DOI:** 10.3389/fpls.2016.00720

**Published:** 2016-05-25

**Authors:** Ping Wang, Xun Sun, Xin Jia, Na Wang, Xiaoqing Gong, Fengwang Ma

**Affiliations:** State Key Laboratory of Crop Stress Biology for Arid Areas, College of Horticulture, Northwest A&F UniversityYangling, China

**Keywords:** apple, ATG8, *Arabidopsis*, autophagy, leaf senescence, nutrient deficiency

## Abstract

Nutrient deficiencies restrict apple (*Malus* sp.) tree growth and productivity in Northwest China. The process of autophagy, a conserved degradation pathway in eukaryotic cells, has important roles in nutrient-recycling and helps improve plant performance during periods of nutrient-starvation. Little is known about the functioning of autophagy-related genes (*ATG*s) in apple. In this study, one of the *ATG8* gene family members *MdATG8i* was isolated from *Malus domestica*. MdATG8i has conserved putative tubulin binding sites and ATG7 interaction domains. A 1865-bp promoter region cloned from apple genome DNA was predicated to have *cis*-regulatory elements responsive to light, environmental stresses, and hormones. MdATG8i transcriptions were induced in response to leaf senescence, nitrogen depletion, and oxidative stress. At cellular level, MdATG8i protein was expressed in the nucleus and cytoplasm of onion epidermal cells. Yeast two-hybrid tests showed that MdATG8i could interact with MdATG7a and MdATG7b. In *Arabidopsis*, its heterologous expression was associated with enhanced vegetative growth, leaf senescence, and tolerance to nitrogen- and carbon-starvation. *MdATG8i*-overexpressing “Orin” apple callus lines also displayed improved tolerance to nutrient-limited conditions. Our results demonstrate that MdATG8i protein could function in autophagy in a conserved way, as a positive regulator in the response to nutrient-starvation.

## Introduction

Plants utilize sophisticated mechanisms for recycling intracellular constituents when nutrient supplies are limited (Doelling et al., 2002). In eukaryotes, two main pathways—the ubiquitin-26S proteasome system and autophagy—are involved in nutrient-recycling, turnover of organelles and aberrant/aggregated proteins, and the precise control of regulators necessary for growth and development (Marshall et al., 2015).

Autophagy is an intracellular degradation system conserved among eukaryotes, with generation of a double-membrane structure (i.e., “autophagosome”) that emerges and sequesters cytoplasmic components as a landmark event (Mizushima et al., 2011), followed by breaking down and recycling cellular materials during development or upon encountering stress conditions (Noda and Inagaki, 2015; Yang and Bassham, 2015). Studies with *Saccharomyces cerevisiae* have identified many autophagy-related proteins (AuTophaGy, or ATGs) that participate in this process (Tsukada and Ohsumi, 1993; Thumm et al., 1994; Klionsky et al., 2003). Among these proteins, several functional groups help form the autophagosome, including the ATG1 kinase complex, ATG2/9/18 transmembrane complex, phosphatidylinositol 3-kinase (PI3K) complex and ATG8/ATG12 conjugation systems (Mizushima et al., 2011; Li et al., 2015; Noda and Inagaki, 2015).

In higher plants, a suite of homologous ATG proteins directs the autophagy process in response to internal and external factors (Li and Vierstra, 2012; Liu and Bassham, 2012; Feng et al., 2014). The ATG5-ATG12 and ATG8-phosphatidylethanolamine (PE) conjugation systems are two essential components of the autophagic system (Li and Vierstra, 2012; Ohsumi, 2014), and *in vitro* reconstitution of them has indicated that the mechanism of autophagy is conserved from yeasts to plants (Fujioka et al., 2008). During conjugation, ATG8 and ATG12 are activated by a common ATP-dependent E1-activating enzyme, ATG7, which subsequently binds them to a conserved cysteine within ATG7 via a thioester linkage and then transfers them to their respective E2-conjugating enzymes, ATG3 and ATG10. The activated ATG12 is connected covalently to ATG5, then the dimeric ATG16 protein self-oligomerizes and interacts with ATG5 within ATG12-ATG5 conjugate, forming a tetrameric complex which serves as the E3-like ligase that conjugates ATG8 to PE (Chung et al., 2010). This ATG8-PE adduct coats expanding phagophores and serves as a docking platform for factors that promote vesicle closure and tonoplast fusion, as well as for receptors that capture specific cargo (Li and Vierstra, 2012; Rogov et al., 2014; Li et al., 2015; Noda and Inagaki, 2015). Through those receptors, autophagy can selectively remove unwanted large protein complexes such as ribosomes (ribophagy; Hillwig et al., 2011) and proteasomes (proteaphagy; Marshall et al., 2015); insoluble protein aggregates (aggrephagy; Zhou et al., 2013); damaged or inactive organelles including mitochondria (mitophagy; Li et al., 2014), chloroplasts (chlorophagy; Ishida et al., 2008; Wada et al., 2009), peroxisomes (perophagy; Farmer et al., 2013; Kim et al., 2013; Shibata et al., 2013), and endoplasmic reticulum (ER-phagy; Bernales et al., 2007); or invading pathogens (xenophagy; Gutierrez et al., 2004; Nakagawa et al., 2004). In addition to its decisive role in selective autophagy, ATG8 protein and its homologs in mammals and plants have been used as very reliable markers for monitoring autophagic activity, since they are localized to the isolated membranes and autophagosomes (Ichimura et al., 2000). In *Arabidopsis*, all nine ATG8 isoforms display high sequence similarity to yeast ATG8 and have a conserved glycine residue at their C-terminus (Doelling et al., 2002; Hanaoka et al., 2002; Yoshimoto et al., 2004). Among them, however, AtATG8h and AtATG8i differ from the others because they lack extra residues that follow this glycine residue (Avin-Wittenberg et al., 2011), as well as less studied than others.

Plant autophagy has several possible roles in seed development and germination, photomorphogenesis, pathogen resistance, senescence, protections against the negative effects of stress, and nutrient-recycling under starvation conditions (Thompson and Vierstra, 2005; Liu and Bassham, 2012; Hanamata et al., 2014). Autophagy is the major contributor to cellular housekeeping and the reuse of nutrients for alleviating nutrient stress (Li and Vierstra, 2012; Liu and Bassham, 2012; Ohsumi, 2014). In *Arabidopsis*, most *ATG* genes are transcriptionally up-regulated by nutrient-starvation and during leaf senescence (Doelling et al., 2002; Rose et al., 2006; Thomas, 2013). Loss-of-function autophagy mutants are hypersensitive to nitrogen (N)- and fixed-carbon (C)-limiting conditions, and plants show accelerated senescence even under nutrient-rich conditions (Hanaoka et al., 2002; Phillips et al., 2008). Autophagy is active in N-remobilization under either starvation conditions or normal growth (Guiboileau et al., 2012, 2013; Xia et al., 2012; Li et al., 2015). Therefore, all of these reports indicate that autophagy is associated with nitrogen metabolism and plant yields.

Apple (*Malus domestica* Borkh.) is one of the most economically important fruit trees grown worldwide. In China, the major region for apple fruit production is within the Northwest Loess Plateau. However, nutrient deficiencies are a challenge there because of reduced soil fertility and a lack of sufficient rainfall. Given that autophagy is critical for maintaining cell homeostasis, plant vitality, and yields under nutrient-starvation conditions, and ATG8s proteins play key roles in autophagy, we isolated one of the *ATG8*s gene, *MdATG8i*, from *M. domestica*. We further characterized *MdATG8i* gene through expression analysis, promoter isolation and analysis, yeast two-hybrid (Y2H), subcellular localization, and heterologous expression in *Arabidopsis* and “Orin” apple callus. The overexpression analysis demonstrated its functions in response to nutrient stress, showing its potential for breeding crops with improvement toward nutrient-starvation.

## Materials and methods

### Apple plant materials and treatments for gene cloning and expression analysis

Two-year-old plants of apple (*M. domestica* Borkh. “Golden Delicious”), grafted onto rootstock *M. hupehensis*, were grown in pots in the greenhouse at the Horticultural Experimental Station of Northwest A&F University, Yangling, China. Standard horticultural and management practices were followed for disease and pest control.

To analyze the tissue-specific expression of *MdATG8i*, buds and flowers were collected on 20 March and 15 April 2014, while stems, shoots, and roots were sampled on 6 June 2014. The fruits were collected 150 days after blooming (15 September 2014). Mark the leaves after they emerged. Young, mature, and senescent leaves were collected on 1 May, 1 August, and 30 October 2014, respectively.

To examine the expression of *MdATG8i* under N-starvation, we applied N-starvation to hydroponically grown seedlings of *M. hupehensis* Rehd. (Bai et al., 2008; Li et al., 2012). For this treatment, the Ca(NO_3_)_2_ and KNO_3_ in our Hoagland's nutrient solution were replaced by CaCl_2_ and KCl, respectively, while the control plants continue to receive the standard solution. Leaves and white roots were sampled on Days 0, 2, 4, 6, and 8 after treatments. To induce oxidative stress, we supplemented the nutrient solution with 50 mM methyl viologen (MV) and collected the white roots at 0, 3, 6, 9, 12, 24, and 36 h post-treatments.

### RNA extraction, cloning, and quantitative real-time PCR

Total RNA was extracted according to a CTAB method (Chang et al., 1993). Residual DNA was removed by treating with RNase-free DNase I (Invitrogen, Carlsbad, CA, USA). First-strand cDNA was generated by using a RevertAid^*TM*^ First Strand cDNA synthesis kit (Fermentas, Thermo Scientific, Waltham, MA, USA). The coding sequence (CDS) of *MdATG8i* was amplified from cDNA with gene-specific primers (Table 1) using high fidelity Platinum^®^ *Taq* DNA Polymerase (Invitrogen).

**Table 1:** **Primers used in this study**.

**Primer**	**Purpose/Vector**	**Sequence (5′–3′)**
*MdATG8i*–F	Clone/pMD19T–simple	F: ATGGGGAAGATCCAATCTTTCAAG
*MdATG8i*–R		R: TTAGCCAAAGGTTTTCTCGCTGCT
BK–*MdATG8i*–*Eco*R I –F	Yeast two–hybrid/pGBKT7	F: CCGGAATTCATGGGGAAGATCCAATC
BK–*MdATG8i*–*Bam*H I –R		R: CGCGGATCCTTAGCCAAAGGTTTTC
AD–*MdATG7a*–*Eco*R I –F	Yeast two–hybrid/pGADT7	F: CCGGAATTCATGGAGGGAGGC
AD–*MdATG7a*–*Cla* I –R		R: CCATCGATTCAAACTTCAACCAAATCATC
AD–*MdATG7b*–*Eco*R I –F		F: CCGGAATTCATGGAGCGAGGC
AD–*MdATG7b*–*Cla* I –R		R: CCATCGATTCAAACCTCAACCAAATCATC
pro–*MdATG8i*–F	Promoter clone/pMD19T–simple	F: GAACAACAAACAATGATTATTTGACA
pro–*MdATG8i*–R		R: GATTGGATCTTCCCCATAAAGC
OE–*MdATG8i*–F	Plant overexpression and Subcellular localization /pGWB406–GFP	F: AAAAAAGCAGGCTTCATGGGGAAGATCCAATC
OE–*MdATG8i–*R		R: CAAGAAAGCTGGGTTTTAGCCAAAGGTTTTCTC
q *MdATG8i*–F	qRT–PCR	F: GCAGCAGGCTTCACTTGACTCC
q *MdATG8i*–R		R: GGAATCCATGCGACTGGCTGTT
*AtUBQ10*–F		F: TTCACTTGGTCCTGCGTCTTC
*AtUBQ10*–R		R: CATCAGGGATTATACAAGGCCCC
attB1	Adapter primer for gateway vector	F: GGGGACAAGTTTGTACAAAAAAGCAGGCT
attB2		R: GGGGACCACTTTGTACAAGAAAGCTGGGT

Restriction sites and adapters are underlined.

The same amount of mRNA (2 μg) from each sample was used to synthesize first-strand cDNA. qRT-PCR was performed on an iQ 5.0 instrument (Bio-Rad, Hercules, CA, USA), using a SYBR Premix Ex Taq kit (TaKaRa, Kyoto, Japan) according to the manufacturer's instructions. Transcripts of the *Malus* elongation factor 1 alpha gene (*EF-1*α; DQ341381) were used to standardize the cDNA samples for different genes. The specific primer sequences for expression analysis are shown in Table 1. The experiments were performed using three biological samples with three technical PCR repeats for each RNA extract.

### Genomic DNA extraction and *MdATG8i* promoter isolation

Apple genomic DNA was extracted from mature leaves of “Golden Delicious” using an improved CTAB method (Modgil et al., 2005). The DNA with good purity and quality was used to amplify the *MdATG8i* promoter. PCR was performed with high fidelity Platinum^®^ *Taq* DNA Polymerase (Invitrogen) with specific primers (Table 1).

### Analyses of sequences and phylogenetics

Putative conserved domains were predicted from the National Center for Biotechnology Information (NCBI), using the BLASTp program (http://blast.ncbi.nlm.nih.gov/Blast.cgi). Homologous sequences from other species were aligned with the ClustalW program (Thompson et al., 1994). Phylogenetic trees were constructed with MEGA 5.0 software (Tamura et al., 2011), using the Neighbor-Joining method and bootstrap tests with 1000 replications. Putative *cis*-regulatory elements in *MdATG8i* promoter region were examined using the PlantCARE web tool (http://bioinformatics.psb.ugent.be/webtools/plantcare/html/).

### Yeast two-hybrid assays

To generate constructs for Y2H assays, we cloned the CDS of *MdATG8i* into the pGBKT7 vector (Clontech Laboratories, Inc., http://www.clontech.com/) to produce a translational fusion with a GAL4 DNA binding domain. Likewise, we separately introduced the CDS of *MdATG7a* (KF438034) and *MdATG7b* (KF438035) into the pGADT7 vector (Clontech) to produce a translational fusion with a GAL4 activation domain. Primers with restriction sites used for the constructs are listed in Table 1. The constructs were co-transformed into yeast strain AH109 according to the Clontech protocol. Protein–protein interactions were examined by checking the growth of the transformed colonies on SD-Leu-Trp and SD-Leu-Trp-His-Ade+X-α-gal plates.

### Plant-overexpressing vector construction for *MdATG8i*

The Gateway binary vector pGWB406 with a GFP fusion at the N-terminus was used for constructing the *MdATG8i*-overexpressing vector. We followed the Gateway Technology protocol (Invitrogen) and inserted *MdATG8i* into pGWB406 destination vectors by BP and LR reactions, using pDONR222 as the donor vector. These vectors were driven by the CaMV 35S promoter and carried the kanamycin (Kan) selectable marker in plants.

### Subcellular localization analysis of *MdATG8i*

Plasmids of GFP-pGWB406/*MdATG8i* and control were transiently transformed, via the gene gun method (Bio-Rad), into onion epidermal cells. After bombardment, the cells were cultured under darkness for 16–20 h in an MS medium at 22°C before being observed with a confocal microscope (Zeiss LSM).

### Generation and characterization of transgenic *Arabidopsis* overexpressing *MdATG8i*

Plant-overexpressing vectors GFP-pGWB406/*MdATG8i* was introduced into *Agrobacterium tumefaciens* strain EHA105 by electroporation. *Arabidopsis thaliana* ecotype “Columbia” (Col-0) was transformed via the floral dip method (Zhang et al., 2006). Growing conditions for these *Arabidopsis* plants included 22°C, 100 μmol photons m^−2^ s^−1^, 70% relative humidity, and a long-day (LD, 16-h) photoperiod. Transgenic seeds (T_1_) were harvested, surface-sterilized in 50% bleach, and selected on an MS medium supplemented with 50 mg L^−1^ Kan. Kan-resistant plants were PCR-confirmed and further selected for T_2_ homozygous lines. The collected T_3_ seeds from two independent T_2_ lines and the wild-type (WT) were used for the following treatments.

To monitor differences in performance between WT and transgenic *Arabidopsis* plants, we sowed T_3_ seeds in soil and exposed them to a short-day (SD, 10-h) photoperiod. The maximum radius of each basal rosette was measured on alternate days with an electronic digital caliper. Other T_3_ seeds were germinated and grown under LD conditions. Photographs were taken to depict the status of natural flowering and leaf senescence during the experiments.

To analyze bolting time, 5-day-old plate-grown *Arabidopsis* seedlings were transferred to either standard MS or sugar-depleted media, where they were grown under LD conditions. Bolting was scored as the time at which the main inflorescence shoot had elongated to 5 mm long (Hanaoka et al., 2002).

To conduct dark-induced leaf senescence, we detached the first and seconds true leaves from 2-week-old seedlings grown on MS plates under LD conditions, then placed them adaxial side-up (Oh et al., 1996) on papers wetted with 3 mM MES (pH 5.7), and held them at 22°C under darkness (Doelling et al., 2002). Chlorophyll was extracted with 80% acetone and contents were determined spectrophotometrically according to the method of Lichtenthaler and Wellburn (1983).

To study the response of WT and transgenic *Arabidopsis* to nitrogen-starvation, we used 5-day-old seedlings grown under LD and transferred them to MS control, N-depleted, or N/sugar-depleted agar media (Doelling et al., 2002). The N-depleted medium was prepared by replacing KNO_3_ and Ca(NO_3_)_2_ with KCl and CaCl_2_, respectively. After 10 days, the plants were photographed under a light microscope (DM2000; Leica, Germany).

The response of WT and transgenic *Arabidopsis* to C-starvation was evaluated with 7-day-old seedlings that had grown under LD conditions. They were transferred to either MS-only or sugar-depleted agar media. After the plates were wrapped with aluminum foil and incubated in the dark for 10 days, they were returned to LD conditions for 10 days. Following this recovery period, the plants were photographed and their root hairs were observed under a light microscope (DM2000; Leica, Germany). As a second test, T_3_ seeds of the WT and transgenic lines were germinated and held for 14 days on MS solid media with or without 1% sucrose. Afterward, the plates were wrapped with foil and incubated in the dark for another 14 days. And then the plants were placed under LD conditions for recovery and were photographed daily (Chung et al., 2010).

### Generation of *MdATG8i*-transgenic “Orin” apple callus and treatments

Callus of “Orin” apple was used for gene transformation (Li et al., 2002) and growth assays. They were placed in the dark at 25°C on an MS medium containing 1.0 mg L^−1^ 2,4-D, 1.0 mg L^−1^ 6-BA, and 8 g L^−1^ agar, and were sub-cultured at 15-d intervals. For transformation, 7-day-old callus grown in a liquid medium were co-cultivated (10 min, gentle rotation, 25°C) with *A. tumefaciens* EHA105 that carried the GFP-pGWB406/*MdATG8i* plasmid. After 2 days co-cultivation, the callus was washed three times with sterile water containing 400 mg L^−1^ cefotaxime (Cef), then transferred to a subculture medium supplemented with 200 mg L^−1^ Cef and 30 mg L^−1^ Kan for transgene selection (Xie et al., 2012).

To investigated the response of transgenic “Orin” callus and the WT to nutrient deficiencies, we shifted 0.02-g samples of 10-day-old tissue to the following treatment media: (1) MS control (subculture); (2) Low-nitrogen, with the N concentration decreased from 60 to 5 mM; or (3) Low-carbon, with the sucrose concentration reduced from 30 to 3 g L^−1^. All plates were placed in the dark at 25°C, and the callus was photographed and weighed on Day 20.

### Statistical analysis

The treatments were repeated three times with consistent results. Data from one representative experiment are shown here, expressed as means and standard deviation (SD). Statistical differences were compared by Student's *t*-tests at significance levels of ^*^P < 0.05, ^**^P < 0.01, or ^***^P < 0.001.

## Results

### Molecular cloning and sequence analysis of *MdATG8i*

**Figure 1:**
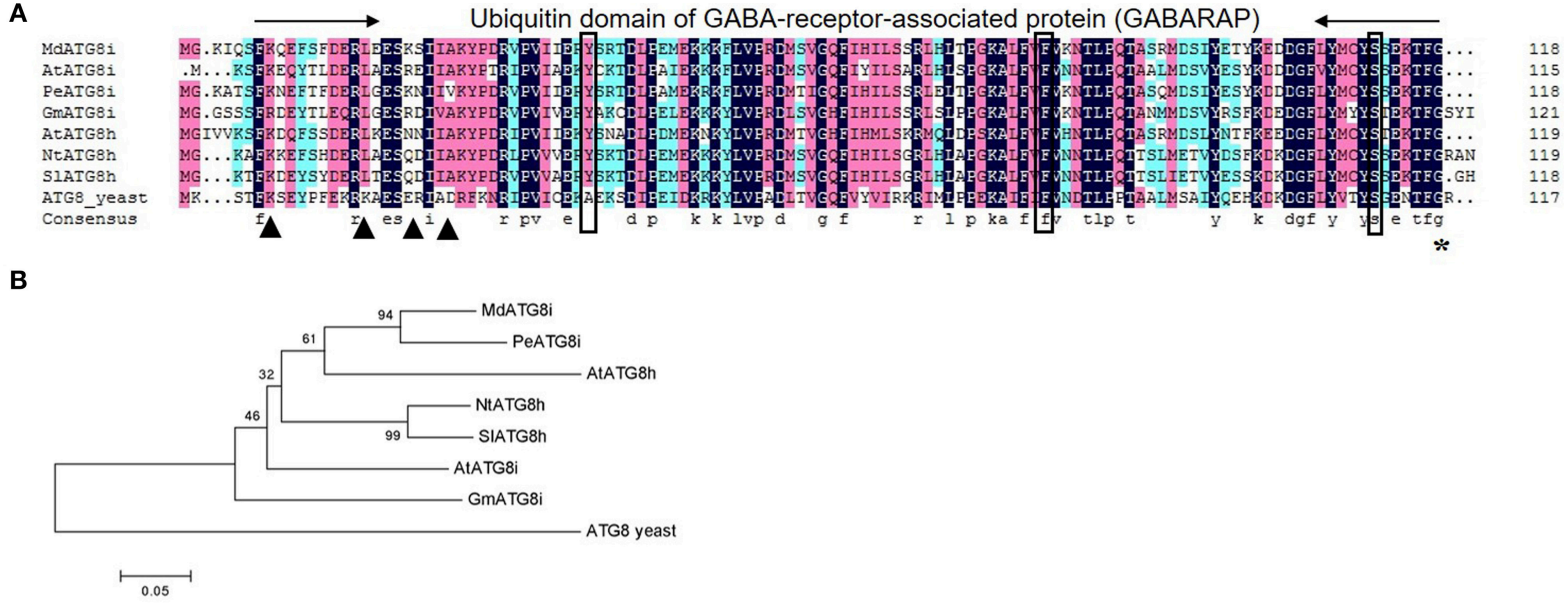
**Analysis of amino acid sequence of *MdATG8i*. (A)** Alignment of deduced amino acid sequence from *MdATG8i* with other *ATG8h* and *ATG8i* members. Black triangle, putative tubulin binding sites; Box, ATG7 binding sites; *, Conserved glycine residue; **(B)** Phylogenetic analysis of ATG8 proteins from *Malus domestica* (*Md*), *Arabidopsis thaliana* (*At*), *Populus euphratica* (*Pe*), *Glycine max* (*Gm*), *Nicotiana tabacum* (*Nt*), *Solanum lycopersicum* (*Sl*), and *Saccharomyces cerevisiae* (yeast). GenBank Accession Numbers are listed in Table 2.

We identified a homologous sequence of *AtATG8i* from genomic database of *M. domestica* “Golden Delicious” (GenBank Accession No. KF438037), named as *MdATG8i*. The gene contains a 357-bp open reading frame (ORF) and encodes a deduced protein with 118 amino acids. Predictions of the conserved domains showed that it has putative tubulin binding sites and ATG7 binding sites, and a conserved ubiquitin domain of GABA-receptor-associated protein (GABARAP) that belongs to the UBQ superfamily (Figure 1A). Protein alignment with other ATG8h or ATG8i from *A. thaliana, Populus euphratica, Glycine max, Nicotiana tabacum,* and *Solanum lycopersicum* (Table 2) showed a 79.33% similarity. Like AtATG8h and AtATG8i, MdATG8i protein lacks extra residues at the C-terminus that follows the conserved glycine residue (Figure 1A). The phylogenetic tree analyses indicated that MdATG8i protein forms a close cluster with PeATG8i and AtATG8h (Figure 1B).

**Table 2:** **GenBank Accession Numbers for proteins used to construct phylogenetic trees**.

**Protein**	**Species**	**GenBank Accession Number**
MdATG8i	*Malus domestica*	AID50965.1
AtATG8h	*Arabidopsis thaliana*	NP_566283.1
AtATG8i	*Arabidopsis thaliana*	NP_566518.1
PeATG8i	*Populus euphratica*	XP_011011529.1
GmATG8i	*Glycine max*	NP_001235960.1
NtATG8h	*Nicotiana tabacum*	AIS71902.1
SlATG8h	*Solanum lycopersicum*	NP_001234639.1
Yeast ATG8	*Saccharomyces cerevisiae*	NP_009475.1

### Expression analysis of *MdATG8i*

**Figure 2:**
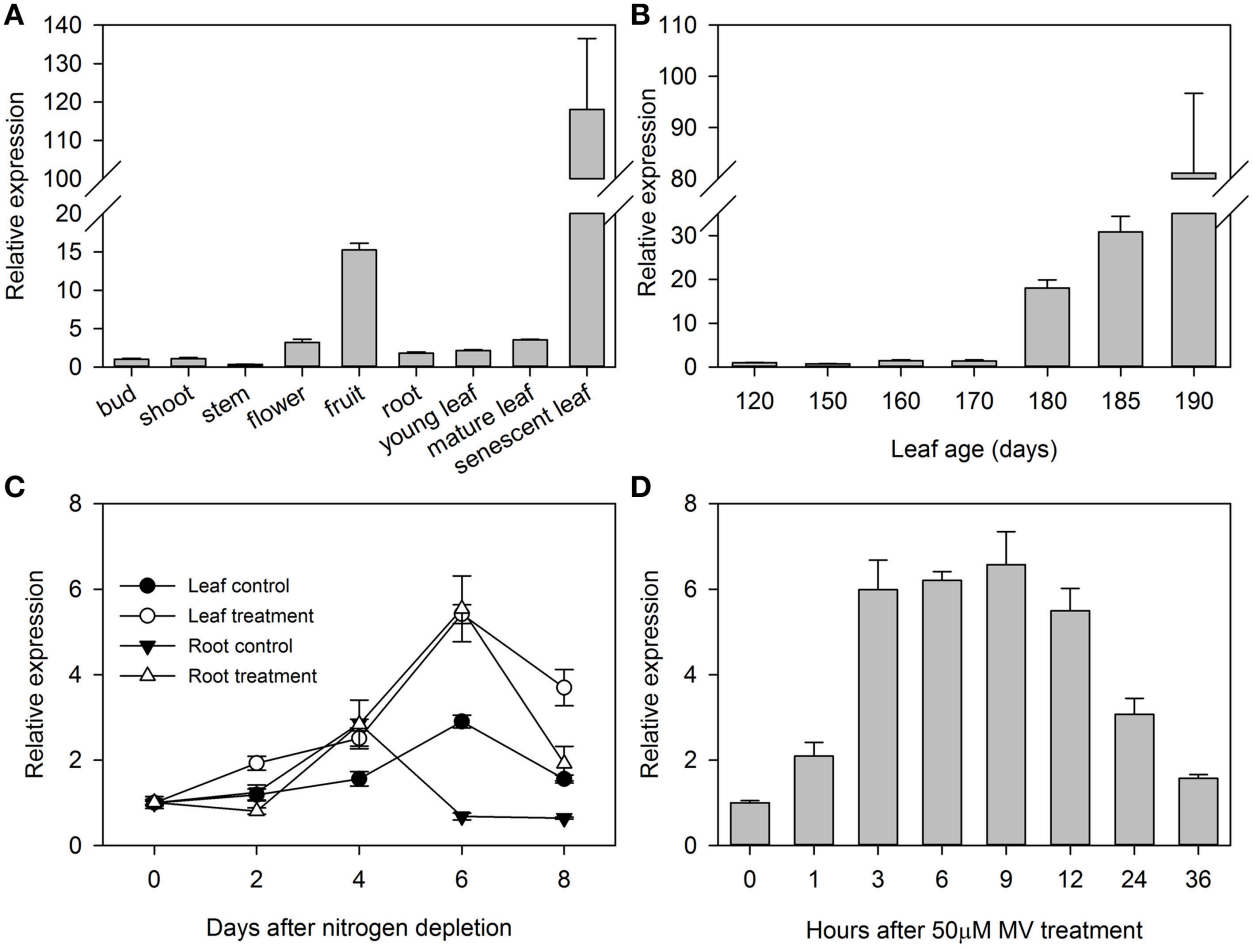
**Expression profiles for *MdATG8i* in different tissues (A), during leaf senescence (B), under nitrogen-starvation (C), and under MV-induced oxidative stress (D)**. Total mRNA was extracted from samples and qRT-PCR was performed with gene-specific primers. Expression levels were calculated relative to expression of *Malus EF-1*α mRNA. Data are means ± SD of three replicate samples.

qRT-PCR analysis showed that *MdATG8i* transcripts were detected in the buds, shoots, stems, flowers, roots, young and mature leaves, and were especially highly accumulated in the fruits and senescent leaves (Figure 2A). Expression in the leaves was greatly enhanced upon aging (Figures 2A,B). To monitor patterns of expression for *MdATG8i* during the senescence process, we analyzed samples of fully mature leaves over time. *MdATG8i* transcript levels were relatively low from Days 120 to 170, but were significantly up-regulated when leaves began to turn yellow. In 190-day-old leaves, *MdATG8i* expression was up-regulated by 80-fold (Figure 2B).

Here, we used hydroponics culturing system with N-depleted Hoagland's nutrient solution to examine the responses of *MdATG8i* to nitrogen-starvation. It showed that *MdATG8i* expression was differentially induced in the leaves and roots, with respective upregulation by 87% and seven-fold at Day 6 (Figure 2C). Using the same hydroponics system, we found that treatment in the roots with an oxidative stress-inducer MV, stimulated gradual upregulation of *MdATG8i* in the first 3–12 h, with a 5.6-fold increment at 9 h (Figure 2D). Therefore, the results demonstrated that *MdATG8i* was responsive to leaf senescence, nitrogen-depletion, and oxidative stress.

### *MdATG8i* promoter sequence analysis

**Figure 3:**
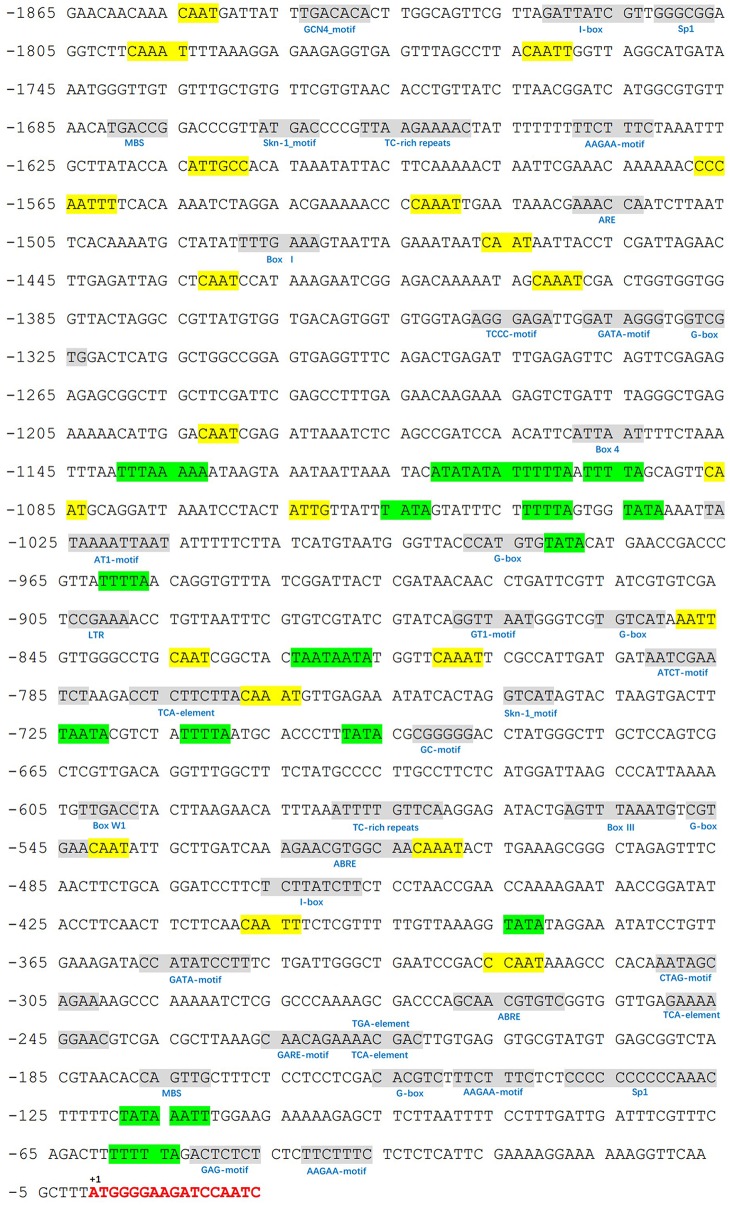
**Putative *cis*-regulatory elements (shaded in gray) in promoter region of *MdATG8i*, as predicted from PlantCARE database**. A 1865-bp fragment was cloned from apple genome DNA, with a GenBank Accession Number KU379689. Key sequence elements—TATA box and CAAT box—are shaded in green and yellow, respectively. Letters in red indicates the coding sequence of *MdATG8i*.

We identified a 1865-bp fragment (GenBank Accession No. KU379689) from the upstream of *MdATG8i* CDS region. Analyses of potential regulatory *cis*-acting elements by PlantCARE program and revealed that *MdATG8i* promoter contains typical CAAT and TATA boxes, as well as many light-responsive elements, including Box I, G-box, ATCT-motif, Box 4, GT1-motif, Sp1, GATA-motif, I-box, TCCC-motif, GAG-motif, and AT1-motif (Figure 3; Table 3). There were also MBS, TC-rich repeats, Box-W1, ARE, GC-motif, and LTR that involved in responses to environmental stresses (Figure 3; Table 3). Beyond that, some important *cis*-regulatory elements in responsive to hormones were also identified, such as ABRE involved in the abscisic acid responsiveness, gibberellin-responsive GARE-motif, salicylic acid-responsive TCA-element, and auxin-responsive TGA-element (Figure 3; Table 3). In this promoter region, there were also a protein binding site Box III, and two motifs (GCN4 and Skn-1) required for endosperm expression (Figure 3; Table 3). These analyses suggested that *MdATG8i* might be regulated by various factors, such as light, environmental stresses and hormone signaling.

**Table 3:** **Predicted *cis*-regulatory elements with putative functions identified in promoter of *MdATG8i* using PLANTCARE database**.

***Cis*-element**	**Positions**	**Sequences**	**Putative function**
MBS	185 (−)	CGGTCA	MYB binding site involved in drought-inducibility
	1689 (−)	CAACTG	
TC-rich repeats	208 (−)	GTTTTCTTAC	Element involved in defense and stress responsiveness
	1286 (+)	ATTTTCTTCA	
ABRE	1341 (+)	AGTACGTGGC	Element involved in the abscisic acid responsiveness
	1344 (+)	ACGTGGC	
	1597 (+)	GCAACGTGTC	
GARE-motif	1640 (−)	TCTGTTG	Gibberellin-responsive element
TCA-element	1088 (−)	TCAGAAGAGG	Involved in salicylic acid responsiveness
	1614 (+), 1643 (+)	CAGAAAAGGA	
TGA-element	1648 (+)	AACGAC	Auxin-responsive element
Box III	1307 (−)	CATTTACACT	Protein binding site
Box-W1	1263 (+)	TTGACC	Fungal elicitor responsive element
ARE	227 (−)	TGGTTT	Regulatory element essential for the anaerobic induction
GC-motif	1173 (−)	CCCCCG	Enhancer-like element involved in anoxic specific inducibility
LTR	962 (+)	CCGAAA	Involved in low-temperature responsiveness
GCN4_motif	22 (−)	TGTGTCA	Involved in endosperm expression
Skn-1_motif	184 (−), 199 (−), 1016 (+), 1121 (+)	GTCAT	Regulatory element required for endosperm expression
Box I	377 (−)	TTTCAAA	Light responsive
G-box	537 (−), 1316 (−), 1006 (−)	CACGAC	Light responsive
	1343 (−), 1599 (−)	CACGTT	
	1710 (+)	CACGTC	
	877 (−)	CACATGG	
ATCT-motif	1074 (+)	AATCTAATCT	Light responsiveness
Box 4	707 (+), 845 (+)	ATTAAT	Light responsiveness
GT1-motif	997 (+)	GGTTAAT	Light responsive element
Sp1	54 (+)	GGGCGG	Light responsive element
	532 (−), 1727 (+)	CC(G/A)CCC	
GATA-motif	528 (+)	GATAGGG	Part of a light responsive element
	1509 (−)	AAGGATAAGG	
I-box	44 (−)	GATAATC	Part of a light responsive element
	527 (+)	GATAAGGTG	
	524 (−)	CATATCCAAT	
	1400 (−)	AGATAAGA	
	1509 (−)	GATATGG	
TCCC-motif	518 (−)	TCTCCCT	Part of a light responsive element
GAG-motif	1814 (−)	AGAGAGT	Part of a light responsive element
AT1-motif	839 (−)	ATTAATTTTACA	Part of a light responsive module
CAAT-box	In yellow shade	CAAAT/CCAAT/CAAT	Common cis-acting element in promoter and enhancer regions
TATA-box	In green shade	TATA	Core promoter element around −30 of transcription start

### Interaction of MdATG8i with MdATG7s in yeast

**Figure 4:**
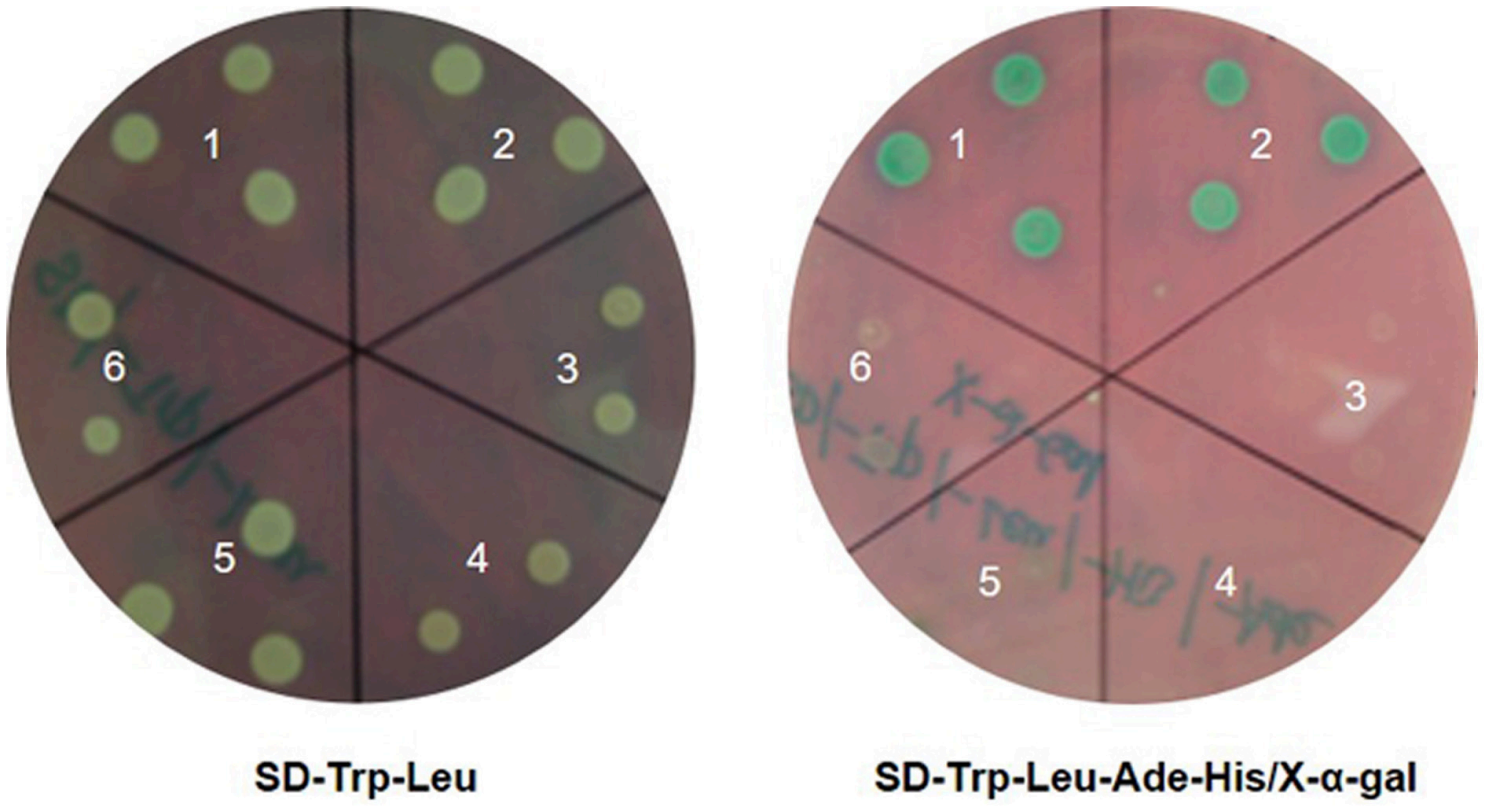
**Yeast two-hybrid assays of full-length *MdATG8i* and MdATG7s proteins**. For testing protein–protein interactions, transformed yeast cells were grown on selective SD medium lacking Trp and Leu (Left) or SD medium lacking Trp, Leu, Ade, and His nutrients (Right). Empty vectors pGBKT7 and pGADT7 and their combinations with MdATG8i and MdATG7s were used as negative controls. Combinations: 1, MdATG8i BK + MdATG7a AD; 2, MdATG8i BK + MdATG7b AD; 3, MdATG8i BK + AD; 4, BK + MdATG7a AD; 5, BK + MdATG7b AD; and 6, BK + AD.

In yeast, protein ATG8-ATG7 interaction complex was detected *in vitro* and residues involved in this interaction were also structurally examined (Hanada et al., 2009; Taherbhoy et al., 2011). We found that MdATG8i protein has putative ATG7 binding sites (Figure 1A) as well as the active site Cys-575 within MdATG7s. To test whether MdATG8i interacts with MdATG7s, we conducted Y2H assays using *MdATG7a* (KF438034) and *MdATG7b* (KF438035) as prey inserts and *MdATG8i* as the bait insert. Our data indicated that MdATG8i could interact with both in yeast (Figure 4), thereby implying that MdATG8i protein might participate in the ATG8-PE conjugation system in a conserved way in *Malus*.

### Subcellular localization of MdATG8i protein

**Figure 5:**
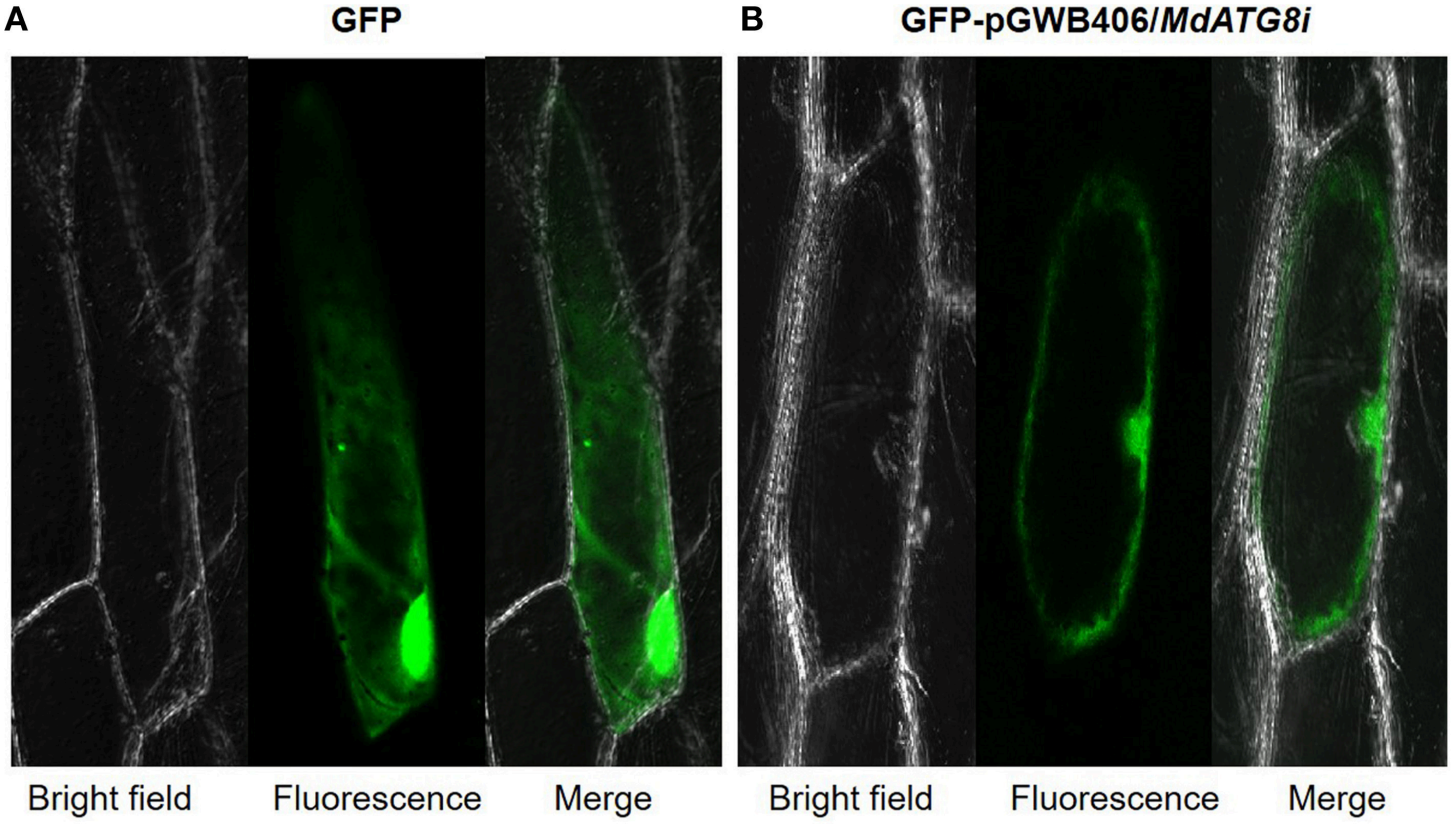
**Subcellular localization analysis of *MdATG8i* in onion epidermal cells. (A)** GPF control; **(B)** MdATG8i-GFP fusion protein.

Driven by the 35S promoter, MdATG8i protein with N-terminus GFP fusion was constitutively expressed in the nucleus and cytoplasm of onion epidermal cells after gene gun bombardment (Figure 5).

### Constitutive expression of *MdATG8i* promoted vegetative growth, bolting, and leaf senescence in transgenic *Arabidopsis*

**Figure 6:**
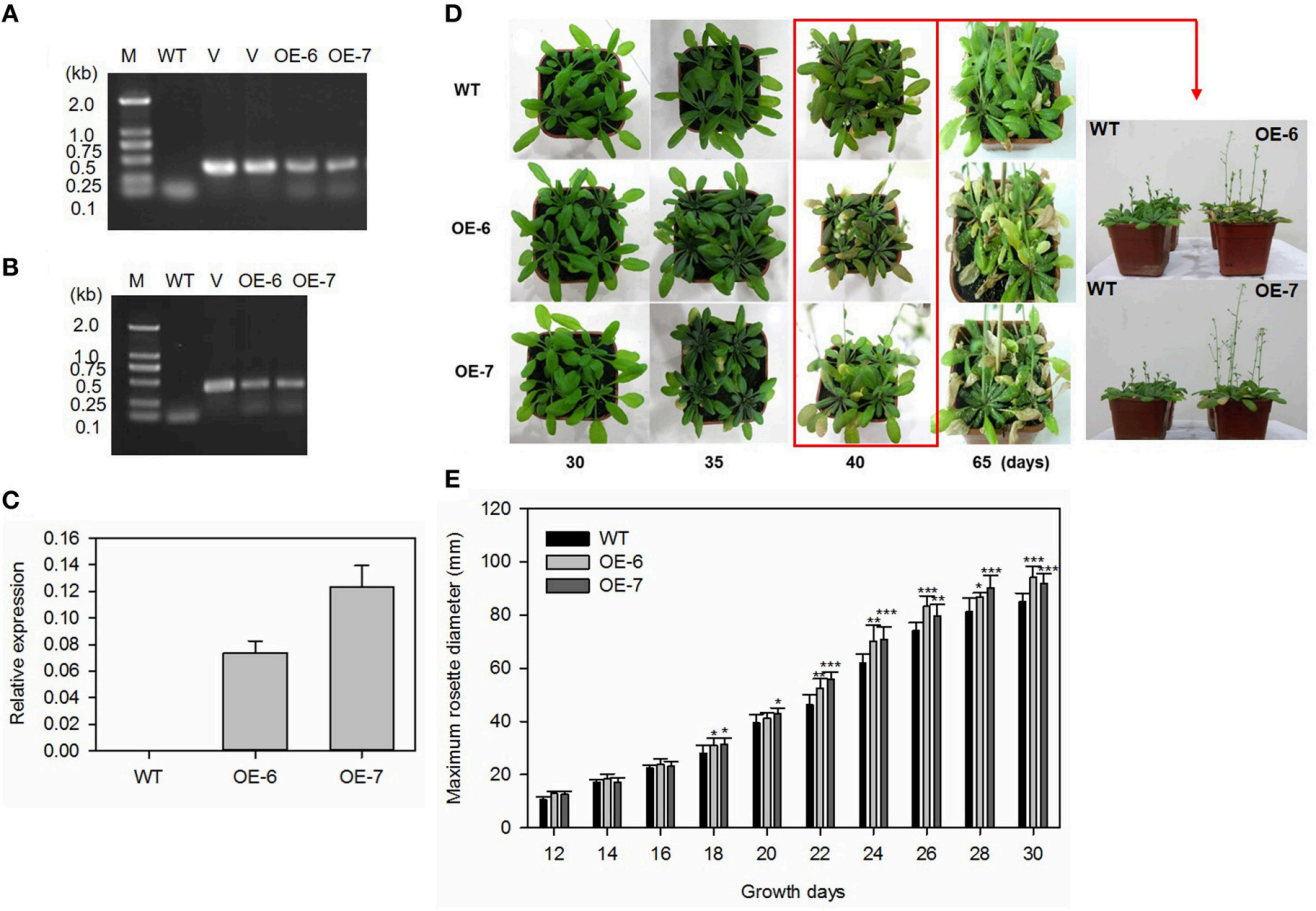
**Heterologous expression of *MdATG8i* in *Arabidopsis* and growth phenotype. (A)** PCR with gDNA; **(B)** PCR with cDNA; Lanes M, molecular marker DL2000; V, positive vector containing pGWB406-*MdATG8i* plasmid; WT, non-transformed wild-type; OE-6 and -7, *MdATG8i*-transgenic lines; **(C)** qRT-PCR analysis of *MdATG8i* transcripts in *Arabidopsis* lines OE-6 and OE-7; **(D)** Growth phenotype comparison of WT and transgenic lines under LD photoperiod; **(E)** Growth for rosettes from WT or transgenic lines under SD photoperiod; Data are means ± SD of 20 replicates. *, ** or *** indicates the statistically significant differences determined by Student's *t*-tests at *P* < 0.05, *P* < 0.01, or *P* < 0.001, respectively.

The construct GFP-pGWB406/*MdATG8i* was introduced into *Arabidopsis* by the floral-dip method (Zhang et al., 2006). Two representative T_2_ homozygous overexpression lines (OE, #6 and #7) with relatively higher expression of *MdATG8i* were selected for further analyses (Figures 6A–C).

Under LD conditions, all of the transgenic plants bolted earlier and had higher main inflorescence height than the WT (Figure 6D). By Day 35, some leaves from transgenic plants were yellowing and beginning to senesce (Figure 6D). As a growth parameter, we measured the maximum rosette radius of *MdATG8i* transgenic *Arabidopsis* plants and the WT under SD conditions. During the first 12–16 days, the radius values did not differ significantly among genotypes. However, *MdATG8i* transgenic plants began to grow faster than the WT after Day 18 (Figure 6E).

**Figure 7:**
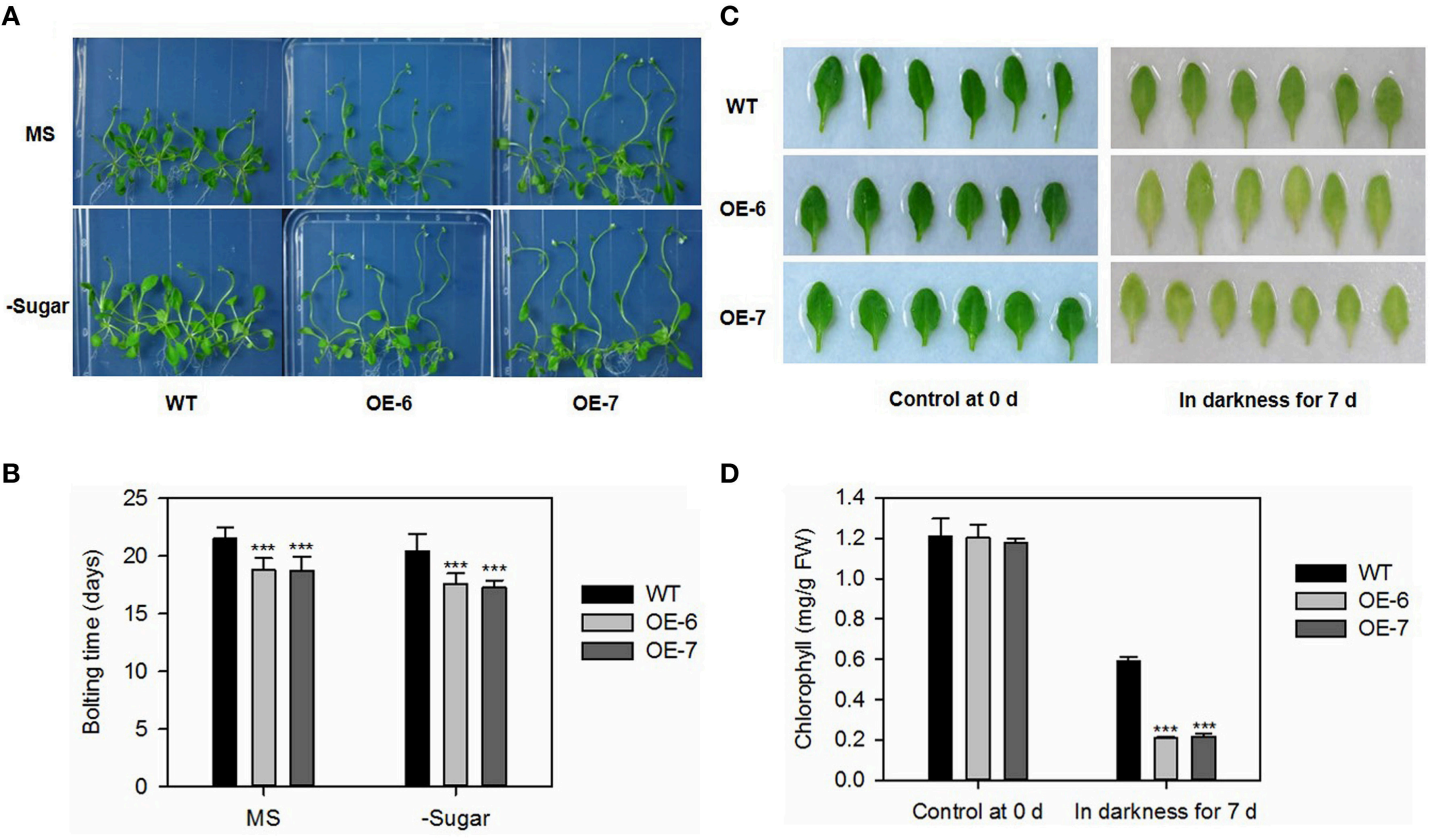
**Bolting and dark-induced leaf senescence is promoted by heterologous expression of *MdATG8i* in *Arabidopsis.* (A)** Bolting comparison between WT and OE lines on Day 25; **(B)** Statistical analysis of bolting time. Data are means ± SD of 18 replicates; **(C)** Representative samples of control and dark-treated tissue from WT and OE lines; **(D)** Chlorophyll contents. Data are means ± SD of five replicates. *, ** or *** indicates the statistically significant differences determined by Student's *t*-tests at *P* < 0.05, *P* < 0.01, or *P* < 0.001, respectively.

To evaluate the difference in bolting days, we transferred 7-day-old *Arabidopsis* seedlings to plates containing either a standard MS medium (Murashige and Skoog, 1962) or one that was sugar-depleted. In both media types, plants of the overexpressing lines were taller, and had bolted significantly earlier than the WT (Figures 7A,B). We also tested the senescence progress of detached leaves under darkness, and found that, after 7 days, the transgenic lines showed earlier yellowing (Figure 7C) and lower chlorophyll contents (Figure 7D) when compared with the WT.

### *MdATG8i* enhanced tolerance to N- and C starvation in transgenic *Arabidopsis*

**Figure 8:**
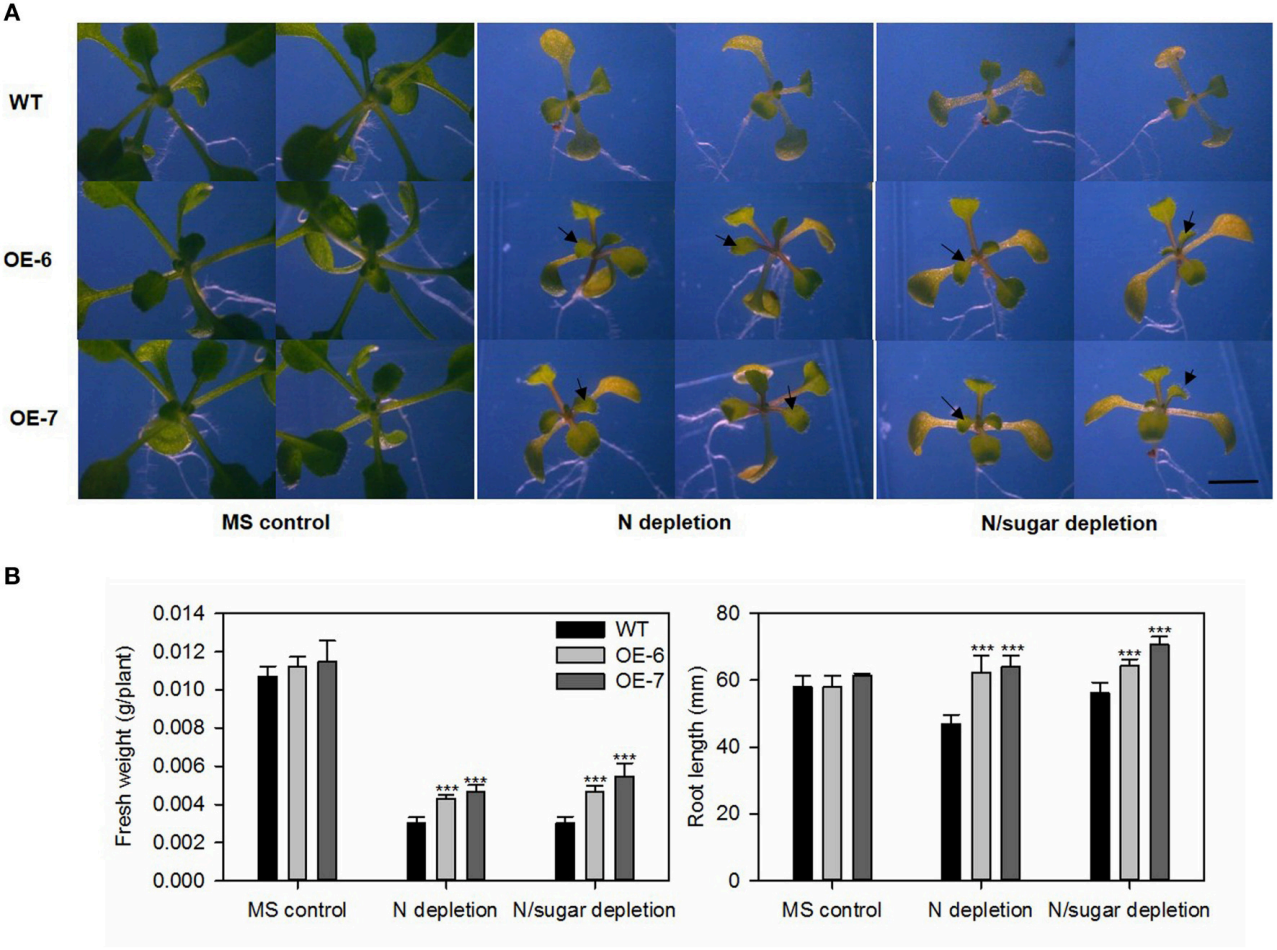
**Tolerance to nitrogen-starvation is enhanced by heterologous expression of *MdATG8i***. Five-day-old *Arabidopsis* seedlings were transferred to MS control, N-depleted, or N/sugar-depleted agar media. After 10 days under LD photoperiod, they were photographed **(A)** before recording fresh weights and root lengths **(B)**. Black arrowheads indicate newly emerged true leaves. Scale bar = 5 mm. Data are means ± SD of 15 replicates. *, ** or *** indicates the statistically significant differences determined by Student's *t*-tests at *P* < 0.05, *P* < 0.01, or *P* < 0.001, respectively.

We transferred 5-day-old *Arabidopsis* seedlings of the transgenic lines and WT into media that were either nitrogen-depleted or both nitrogen- and sugar-depleted. After exposure to LD conditions for 10 days, the cotyledons of all plants turned yellow. However, we noted distinct differences in the size and growth patterns of the true leaves. As shown in Figure 8A, all transgenic lines produced more and larger true leaves than the WT under both N-depleted and N/sugar-depleted conditions. All OE lines had significantly higher fresh weights and longer roots than the WT after 10 days of growth on N-depleted and N/sugar-depleted media (Figure 8B). This phenotype suggested that, under a nitrogen deficiency, overexpression of *MdATG8i* enables *Arabidopsis* seedlings to perform better than the WT.

**Figure 9:**
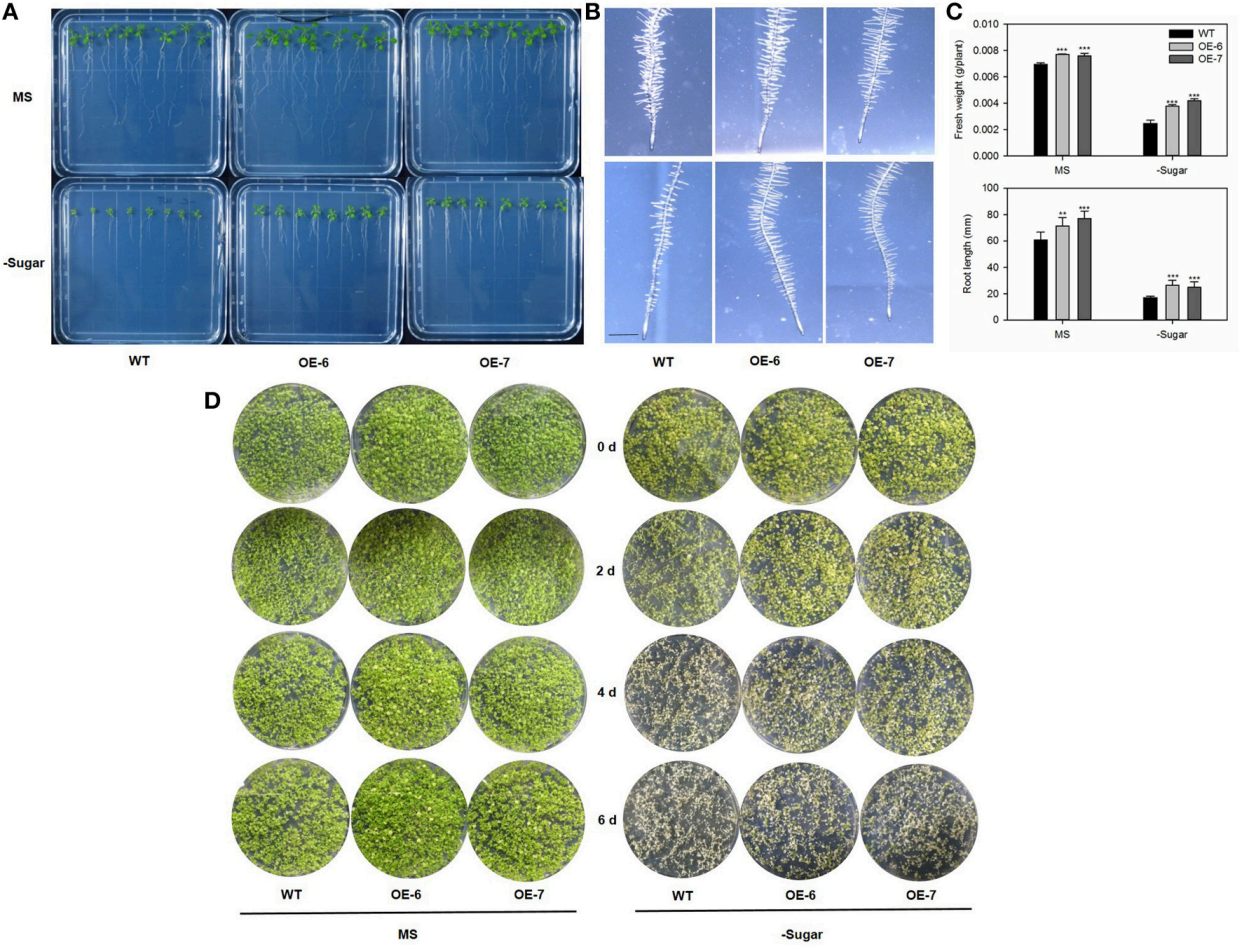
**Results of analysis using plants to test tolerance to carbon-starvation, which is enhanced by heterologous expression of *MdATG8i***. Seven-day-old *Arabidopsis* seedlings were transferred to standard MS or sugar-depleted agar media. After incubation in dark for 10 days, plates were returned to light for recovery under LD photoperiod. **(A)** Seedling phenotypes for WT and OE lines; **(B)** Root hair phenotype; scale bar = 5 mm; **(C)** Fresh weights and root lengths. Data are means ± SD of 15 replicates; **(D)** Alternative analysis using seeds to test tolerance to carbon-starvation. *Arabidopsis* seeds were germinated and grown on MS solid medium with or without 1% sucrose for 14 days. Afterward, plates were incubated in dark for another 14 days. Plants from WT and OE lines were photographed on alternate days (0, 2, 4, 6) to monitor recovery phenotype under LD photoperiod. *, ** or *** indicates the statistically significant differences determined by Student's *t*-tests at *P* < 0.05, *P* < 0.01, or *P* < 0.001, respectively.

Generally, *Arabidopsis atg* mutants are more sensitive to environments in which the supply of carbon is limited (Hanaoka et al., 2002; Thompson, 2005; Phillips et al., 2008). To address whether *MdATG8i*-overexpressing plants have enhanced tolerance to C-starvation, we transferred 7-day-old *Arabidopsis* seedlings to a medium without supplemental sugar and exposed them to continuous darkness for 10 days. Afterward, the plates were returned to the light. Following 10 days of recovery, the transgenic lines had higher fresh weights, more root hair and longer roots, especially when growing on the sugar-depleted medium (Figures 9A–C). It was interesting to find differences in the distribution and density of root hairs. When sugar was omitted from the media, the WT root hairs were sparse and short, while two OE lines showed no obvious variations from those grown on the standard MS medium (Figure 9B). This improved adaptation to carbon-starvation was also confirmed by placing seeds of the *Arabidopsis* WT and transgenic lines directly on either MS or sugar-depleted media for 14 days, then transferring the plates to darkness for 14 days, before allowing them to recover under LD conditions. On the standard media, plants of all genotypes were greener in the light and had produced new leaves, and performance by the transgenic plants was slightly better than that of the WT (Figure 9D). More obvious differences were observed on the sugar-depleted media, where WT plants wilted after 2 days before they quickly turned white and then died. Although some leaves from the transgenic plants also appeared bleached on Day 2, they did not wilt, and some plants survived after 6 days of recovery (Figure 9D). These results demonstrated that overexpression of *MdATG8i* contributes to greater endurance against N- and C-starvation.

### Constitutive expression of *MdATG8i* enhanced tolerance of “Orin” apple callus to limited nutrients

**Figure 10:**
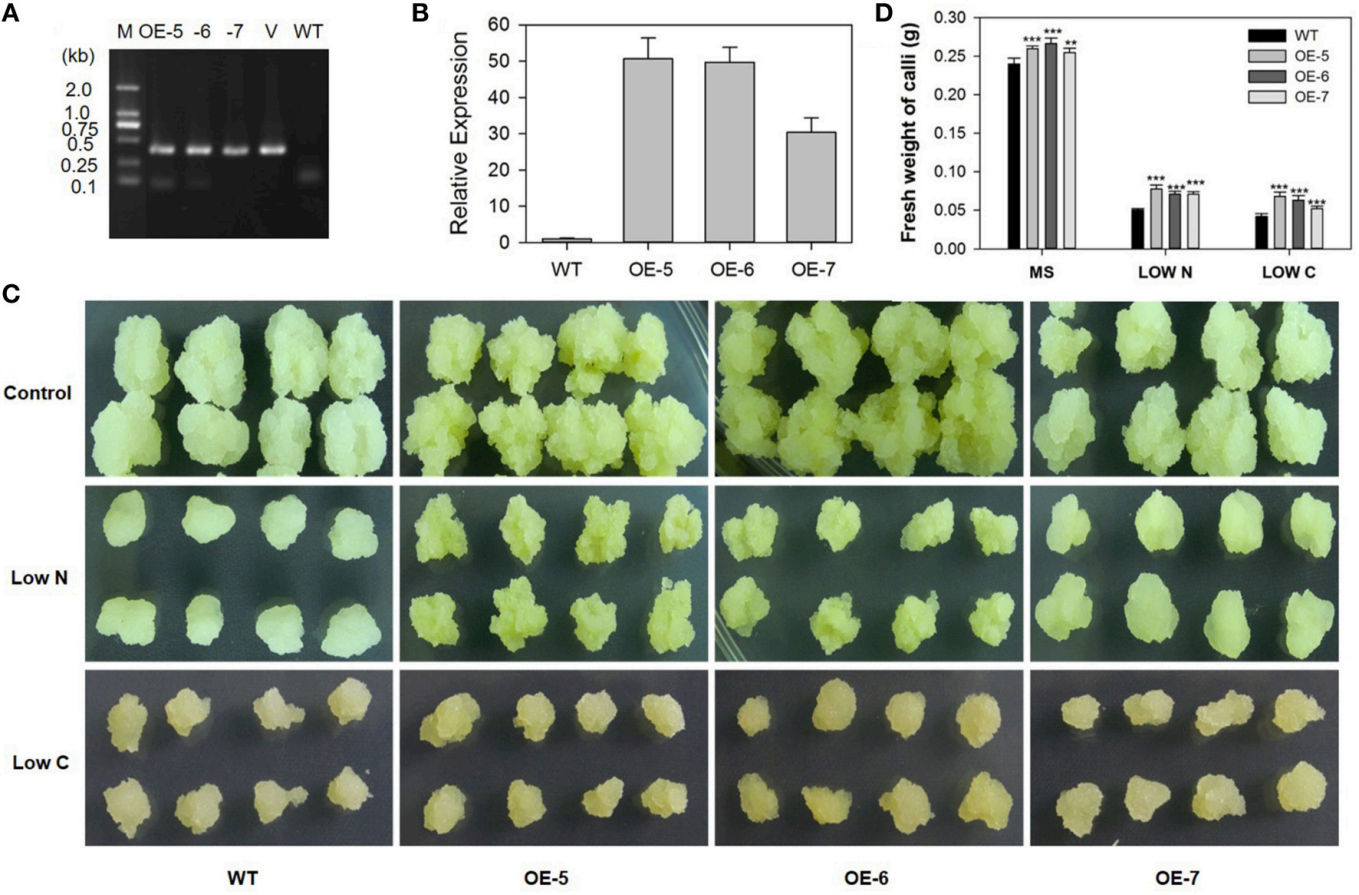
**Tolerance to limited supplies of nitrogen and carbon is enhanced by constitutive expression of *MdATG8i* in transgenic “Orin” apple callus**. **(A)** PCR verification of constitutive expression. Lanes M, molecular marker DL2000; V, positive vector containing pGWB406-*MdATG8i* plasmid; WT, non-transformed wild-type; and OE-5 through -7, *MdATG8i*-transgenic callus lines; **(B)** qRT-PCR analysis of *MdATG8i* expression in WT and transgenic lines; **(C)** Morphology of callus after 20 days; **(D)** Fresh weights of callus after 20 days. Data are means ± SD of three biological replicates. *, ** or *** indicates the statistically significant differences determined by Student's *t*-tests at *P* < 0.05, *P* < 0.01, or *P* < 0.001, respectively.

We obtained three transgenic “Orin” lines that over-express *MdATG8i*, as confirmed by PCR with gDNA (Figure 10A) and by qRT-PCR with cDNA (Figure 10B). The mRNA transcripts were increased by 49-, 48-, and 29-fold in OE-5, OE-6, and OE-7, respectively (Figure 10B). After 14 days of growth, transgenic and WT callus of similar size and weight were transferred to MS control, low-N, or low-C media. By Day 20, the WT callus on the low-N medium had turned white and stopped growing while the three transgenic lines were slightly yellow but showed better growth (Figure 10C). Although all of the callus grown on the low-C medium turned brown (Figure 10C), transgenic callus was significantly heavier than the WT under all treatments (Figure 10D). These results showed that overexpression of *MdATG8i* confers tolerance to nitrogen and sucrose deficiencies at the cellular level.

## Discussion

In response to low energy resources, eukaryotic cells initiate autophagy (Mizushima and Levine, 2010). During that degradative process, autophagosomes are formed. They then engulf a portion of the cytosol or organelles and fuse with a lysosome or vacuole before the cellular cytosolic contents are ultimately recycled (Nakatogawa et al., 2009). MdATG8i protein has the typical conserved glycine residue at the C-terminus. Moreover, this protein has putative tubulin binding sites, thereby suggesting that it is also involved in linking the autophagy pathway to the microtubule network, similar to what happens with AtATG8a and AtATG8d from *Arabidopsis* (Ketelaar et al., 2004). The MdATG8i protein has ATG7 binding sites as well. During autophagy, a thioester-bonded ATG7-ATG8 intermediate has been generated and could be detected *in vitro* (Hanada et al., 2009; Noda et al., 2011). We used Y2H assays to show that MdATG8i could interact with both MdATG7a and MdATG7b in yeast. This was evidence that MdATG8i protein can also participate normally in the ATG8-PE conjugation system, and the essential residues of MdATG8i were well conserved in *M. domestica*.

As the final stage in leaf development, senescence is characterized by chlorophyll degradation and the transition of nutrients from assimilation to remobilization (Masclaux et al., 2000). Autophagy might play important roles in degradation/remobilization during natural or stress-induced leaf senescence (Avila-Ospina et al., 2014; Li et al., 2015). Many *ATG*s in *Arabidopsis* are strongly up-regulated during natural-, dark-induced, and detachment-induced leaf senescence, thereby indicating that autophagy is critical for the efficient remobilization and recycling of nutrients (van der Graaff et al., 2006). The mRNA for ATG8 are preferentially accumulated as leaves senesce, suggesting that the autophagy conjugation pathways are up-regulated during that plant stage (Doelling et al., 2002). We found that transcriptional level for *MdATG8i* is much higher in senescent leaves than in any other tissues examined here. Moreover, it is sharply up-regulated in the late stages of natural senescence, implying that, *MdATG8i* participates in this process (Wang et al., 2014). Because chloroplast degradation involves autophagy (Ishida et al., 2008; Wada et al., 2009), most *atg* mutants from *Arabidopsis* exhibit a phenotype of accelerated and early senescence when compared with the WT (Doelling et al., 2002; Hanaoka et al., 2002; Xiong et al., 2005), a phenomenon thought to be due to excess production of salicylic acid (Yoshimoto et al., 2009). In our study, however, heterologous expression of *MdATG8i* in *Arabidopsis* led to a slight promotion of leaf senescence and an early transition into the reproductive growth stage. Although these phenotypes seem to be inconsistency with those reported for the *atg* mutants, they are similar to those observed when soybean *GmATG8c* is expressed in *Arabidopsis*. There, vegetative growth is stimulated, as evidenced by the development of larger rosettes and earlier bolting (Xia et al., 2012). In addition, the *atg5* and *atg7* mutants from *Arabidopsis* display a functional stay-green phenotype rather than the yellowing that occurs in the WT when exposed to mild abiotic stress (Sakuraba et al., 2014). Our results and these previous reports suggest that autophagy has roles in both plant survival as well as mortality depending upon environmental conditions. And this might be a positive strategy to improve nutrient use efficiency by facilitating the remobilization of chloroplast proteins during leaf senescence by MdATG8i.

*Arabidopsis atg* mutants like *atg7-1* and *atg9-1* are hypersensitive to N- and C-starvation (Doelling et al., 2002; Hanaoka et al., 2002). In *Arabidopsis*, most autophagy genes are transcriptionally up-regulated when nutrients are less available (Doelling et al., 2002; Xiong et al., 2005; Rose et al., 2006; Liu and Bassham, 2012). Furthermore, in maize, *ATG* transcripts and ATG8-PE are accumulated when N and C supplies are reduced (Chung et al., 2009). Consistent with this, *MdATG8i* respond to N-starvation through transcriptional upregulation. Furthermore, overexpression of *MdATG8i* in *Arabidopsis* leads to similar phenotypes, i.e., enhanced tolerance to nitrogen and carbon starvations. The transgenic lines not only grow better than the WT under such conditions, but they recover and have increased performance when returned to normal growth practices. In particular, transgenic plants produce more leaves, longer roots, and more root hairs, regardless of whether they are starved or are moved again to optimum conditions. This suggests that heterologous expression of *MdATG8i* contributes to the fitness and survival of nutrient-stressed *Arabidopsis*.

Previous studies have shown that transgenic *Arabidopsis* plants over-expressing *GmATG8c* produce more leaves under extended periods of N- and C-starvation (Xia et al., 2012). Likewise, expression of GFP-*AtATG8f* -HA under the control of the 35S promoter is associated with improved *Arabidopsis* growth when N and fixed-C are limited (Slavikova et al., 2008). Finally, *Arabidopsis* mutants defective in either the ATG8 or ATG12 conjugation system display the same sensitivity to N or fixed-C starvation (Chung et al., 2010). Therefore, the involvement of MdATG8i in autophagy conjugation systems has a positive role in the maintenance of plant fitness and adaptations to nutrient limitations.

For further evaluation of the function of *MdATG8i* in coping with nutrient-starvation, we constitutively over-expressed *MdATG8i* under the control of the 35S promoter in “Orin” callus. Transgenic callus had much better growth and were significantly heavier than the WT under low-N or -C conditions. Our finding is consistent with that of Xia et al. (2012), and confirms that *MdATG8i* functions against nutrient deficiencies at the cellular level. Because of the challenges associated with apple transformation (Seong and Song, 2008), it is still difficult to produce a large number of transgenic apple plants. Based on our current findings, MdATG8i has a physiological function to protect tissues from the negative effects of nutrient-deprivation, at both the cellular and whole-plant levels. Furthermore, our results demonstrate that this gene has potential as a target gene in research programs that focus on improving yields and making plants more adaptable to nutrient-deficient growing conditions.

In summary, we characterized an apple autophagy gene—*MdATG8i*, analyzed its sequence, expression and promoter, and also examined its function in response to nutrient deficiencies in both *Arabidopsis* and apple callus. The improved tolerance to N- and C-starvation by MdATG8i provided evidence that it owns conserved function in apple autophagy process, which might be linked to elevated fitness in apple plants that grow in challenging environment such as nutrient deficiencies.

## Author contributions

PW and FM conceived and designed the experiments; PW and XS performed the experiments; NW, XJ, and XG collected data; PW wrote the manuscript; FM critically revised the article.

### Conflict of interest statement

The authors declare that the research was conducted in the absence of any commercial or financial relationships that could be construed as a potential conflict of interest.

## Abbreviations

ATG: AuTophaGy-related protein
C: carbon
LD: long-day
MV: methyl viologen
N: nitrogen
OE lines: overexpressing lines
SD: short-day
WT: wild-type.
